# Proof-of-Concept Testing of a Real-Time mHealth Measure to Estimate Postural Control During Walking: A Potential Application for Mild Traumatic Brain Injuries

**DOI:** 10.31372/20180304.1027

**Published:** 2018

**Authors:** Hyunhwa Lee, Sungchul Lee, Laura Salado, Jonica Estrada, Jacob White, Venkatesan Muthukumar, Szu-Ping Lee, Sambit Mohapatra

**Affiliations:** a School of Nursing, University of Nevada, Las Vegas, NV, USA; b Department of Computer Science, University of Wisconsin-Whitewater, WI, USA; c College of Arts, Sciences, and Education, Florida International University, FL, USA; d School of Sciences, University of Nevada, Las Vegas, NV, USA; e College of Liberal Arts, University of Nevada, Las Vegas, NV, USA; f Department of Electrical and Computer Engineering, University of Nevada, Las Vegas, NV, USA; g Department of Physical Therapy, University of Nevada, Las Vegas, NV, USA; h Department of Rehabilitation & Movement Science, University of Vermont, VT, USA

**Keywords:** mobile health (mHealth), mild traumatic brain injury (mTBI), postural control, real-time measure

## Abstract

*Background:* Most individuals with mild traumatic brain injury (mTBI) experience post-injury deficits in postural control. Currently available measures of postural control are lab-based or supervised, which may hinder timely symptom assessment for individuals with mTBI, including Asian populations, who do not seek initial screening post-injury. In this proof-of-concept testing study, we introduce a real-time mobile health (mHealth) system to measure postural control during walking. The proposed mHealth system can be used for home-based symptom assessment and management of mTBI.

*Methods:* In our proposed mHealth system, a smartwatch, a smartphone, and a cloud server communicate to measure, collect, and store body balance data in real time. Specifically, we focus on the rotation vector data that have been reported to be the most effective in terms of differentiating balance control during walking across different participants.

*Results:* Constant motion change in four participants (two females and two males; three healthy participants, and one individual with reduced physical mobility) was collected and analyzed. The results of our data analysis show that, compared to healthy participants, the individual was reduced physical mobility had a wider range of motion between right and left, up and down, and forward and backward while walking. We also found that female participants had narrower ranges of right-to-left and up-and-down motions than their male counterparts.

*Conclusions:* Our results highlight the potential of the proposed real-time mHealth system for home-based symptom assessment and management of mTBI, which may benefit Asian and other nonwhite racial minority groups that appear to be more reluctant to access post-acute rehabilitation services.

The past decade has witnessed a rapid increase in the use of wearable mobile sensors in home-based patient monitoring (Kumar et al., 2013; Patrick, Griswold, Raab, & Intille, 2008). This system mostly benefits individuals who do not seek or have access to, or even ignore necessary medical screening following an injury, such as mild traumatic brain injury (mTBI). Despite being termed “mild,” mTBI produces several persistent functional impairments, such as deficits in postural control (Degani et al., 2017), in up to 80% of cases (Katz, White, Alexander, & Klein, 2004; Valovich McLeod & Hale, 2015). Among individuals who avoid necessary medical screening after mTBI, Asian Americans appear to be particularly reluctant to seek rehabilitation services (Dams-O’Connor et al., 2013; McQuistion et al., 2016). In addition, young adults, such as student athletes or active military service members and veterans, typically consider the mTBI diagnosis to be a stigma and/or a hindrance to their return to play or duty and, for that reason, tend to avoid screening (Iverson & Lange, 2011; Peskind, Brody, Cernak, McKee, & Ruff, 2013). Some individuals may also refuse to admit having problems after an mTBI (Centers for Disease Control and Prevention, 2017). As a result, compliance rate of affected individuals in home environments remains low (Venugopalan, Cheng, Stokes, & Wang, 2013).

At present, most widely used assessments to measure postural control are lab-based or supervised. Along with requiring specialized equipment, such assessments typically do not provide real-time feedback. The limitations of conventional assessments of postural control suggest that a home-based and real-time feedback monitoring device or system can be more efficient—specifically, for the following two reasons: (1) a home-based monitoring device or system does not require a visit to a laboratory or clinic for screening; and (2) real-time feedback of the device or system can benefit those individuals who are unable to receive appropriate and timely assessments.

In this context, we tested whether a new unsupervised mobile health (mHealth) system could be used to monitor postural control in real time during walking, so that individuals using this system would not need to visit a clinic or a research lab. We used a proof-of-concept test as a potential implication for the development and application of the new mHealth system. Therefore, our focus was on introducing the structure and main elements of the proposed unsupervised mHealth system which can further mark out this research as potentially extendable and/or scalable for feasibility and efficacy testing (Kendig, 2016). Consisting of a smartwatch and a smartphone, the system targets individuals with mTBI and enables monitoring and collecting real-time walking balance data in a home environment. In addition, the proposed system may contribute to the development of an effective and inexpensive strategy for detecting early signs of neurological deterioration after mTBI.

## mTBI and Postural Control

TBI is characterized by temporary or permanent neurobiological impairments following an injury. In the U.S. alone, nearly 2.8 million people annually sustain a TBI (Taylor, Bell, Breiding, & Xu, 2017), amounting to $76.5 billion in economic impact (Centers for Disease Control and Prevention, 2013). Racial minority groups, including Pacific people (Tauafiafi, 2014), are reported to have a higher incidence and poorer outcomes of TBI than non-Hispanic Whites (Staudenmayer, Diaz-Arrastia, de Oliveira, Gentilello, & Shafi, 2007). Mild TBI (mTBI), which accounts for over 75–80% of all TBI cases (Kay & Teasdale, 2001; National Center for Injury Prevention and Control, 2003), is conventionally diagnosed based on the following criteria: loss of consciousness shorter than 30 min; posttraumatic amnesia shorter than 24 h; and injury scores from 13 to 15 on the Glasgow Coma Scale (American Congress of Rehabilitation Medicine, 1993; Teasdale & Jennett, 1974). Despite being termed “mild,” mTBI causes multifaceted and persistent functional impairments that can severely disturb the quality of life of affected individuals (Seabury et al., 2018), resulting in long-term disability of over 40% of patients and inability to return to work one year post-injury of a further 25% (Langlois, Rutland-Brown, & Wald, 2006). In our recent studies, we found that college students with mTBI, even long after the injury, showed more body sway with larger oscillations during standing balance tests (Carbonar, Dunning, Greenebaum, Mahoney, & Mohapatra, unpublished; Degani et al., 2017; H. Lee et al., 2018). Other relevant findings of our previous research on individuals who had experienced mTBI include poorer accuracy and velocity of rapid eye movements (Danna-Dos-Santos, Mohapatra, Santos, & Degani, 2018), poorer episodic memory (H. Lee et al., 2018), diminished attention and concentration (Carbonar et al., unpublished), and more severe anxiety and sleep disturbance (H. Lee et al., 2018).

Among a wide array of post-mTBI complications, balance performance-related issues are observed in about 20–80% of mTBI cases (Katz et al., 2004; Valovich McLeod & Hale, 2015). mTBI may cause changes in the cortex and brainstem’s reticular formation (Shaw, 2002) which, in turn, results in altered interaction between different parts of sensory inputs (Guskiewicz, 2011), disrupting the normal orientation information to the postural control system. These disruptions of static and/or dynamic balance may lead to postural instability in either the anterior-posterior direction, medial-lateral direction, or both (Guskiewicz, 2011). Specific symptoms of postural instability include impairments of visual, vestibular, or somatosensory orientation, such as dizziness, vertigo, tinnitus, lightheadedness, blurred vision, or photophobia (Ingersoll & Armstrong, 1992). The duration of these symptoms may extend to months or years post-injury. However, since some or most of these symptoms may not be recognized as necessarily associated with mTBI, affected individuals may not admit having issues with balance control or postural instability post-injury (Centers for Disease Control and Prevention, 2017). If, after an injury, the affected individual does not seek immediate balance screening, the impaired balance control associated with such heterogeneous symptoms may be further aggravated, leading to higher risks for sequelae.

## Real-Time mHealth Measure for Postural Control

In previous research, several well-studied objective measures of balance performance for mTBI have been proposed, such as the force-platform postural-stability test (Degani et al., 2017), postural and gait analysis that uses a motion capture system (Venugopalan et al., 2013), and sensory-motor performance analysis that relies on visuomotor equipment to measure speed and accuracy of eye movement that influence body balance (Heitger, Jones, & Anderson, 2008). In general, compared to clinical interviews or self-reported symptom questionnaires that do not ensure appropriate injury identification and post-injury treatment, the approaches outlined above are more objective in terms of their ability to assess body balance control. However, the limitation of these balance assessments is that they require affected individuals to visit a clinical or research laboratory for assessment. Yet, evidence is available showing that some ethnic groups, such as Asian Americans and Native Americans, are less likely to attend rehabilitation programs (Dams-O’Connor et al., 2013; McQuistion et al., 2016). Along with ethnic background, age and professional occupation can also be factors that contribute to individuals’ reluctance to seek clinical or laboratory assessment: specifically, several studies showed that student athletes or active military service members and veterans typically consider the mTBI diagnosis to be a stigma and/or a hindrance to their return to play or duty (Iverson & Lange, 2011; Peskind et al., 2013). Taken together, these unassessed and/or undertreated TBI cases warrant a new and innovative approach that would offer a possibility of reliable and accurate home-based monitoring for individuals who have had a TBI.

Among numerous wearable devices or mobile sensors for home-based patient monitoring (Kumar et al., 2013; Patrick et al., 2008), inexpensive and widely available mobile health (mHealth) methodologies have been extensively used for monitoring gait disturbances associated with chronic diseases (Juen, Cheng, Prieto-Centurion, Krishnan, & Schatz, 2014). Since symptom development and disease progress following mTBI is known to be heterogeneous in terms of types and timelines (Rosenbaum & Lipton, 2012), real-time assessment and feedback can both detect and prevent further functional deterioration, as well as save affected individuals multiple trips to clinics. In this regard, previous research on other chronic diseases, such as epilepsy, Parkinson’s disease, or multiple sclerosis, has demonstrated that real-time data collection and analysis in these conditions can provide effective and timely reminders for patients during treatment, as well as serves as effective tools for feedback (Cancela et al., 2014; Lamont, Daniel, Payne, & Brauer, 2018).

In the present proof-of-concept testing study, we developed and tested a new real-time mHealth system for unsupervised home-based monitoring of postural control during walking. The real-time mHealth system was developed by a multidisciplinary research team (spanning together nurses, engineers, and physical therapists) who have implemented a cycle of continuous feedback to ensure application relevance, acceptability, and usefulness of the proposed system. The present study focuses on introducing the structure and main elements of the mHealth system. The system may serve as an effective and inexpensive strategy (Kumar et al., 2013) to detect early signs of neurological deterioration after an mTBI, as well as help initiate timely and appropriate treatment and evaluate the rehabilitation process.

## Methods

### Proof-of-Concept Testing of the Real-Time mHealth Measure of Postural Control During Walking

**Figure 1:**
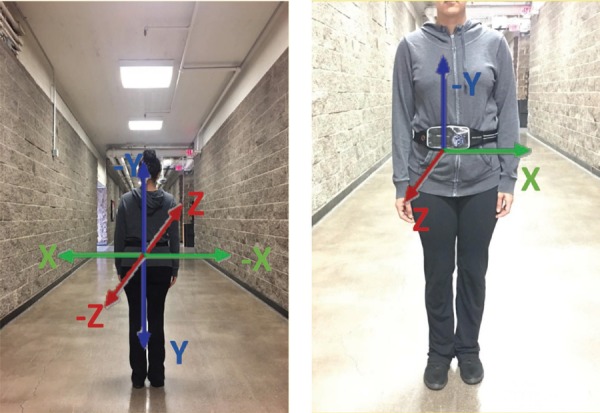
XYZ-axis orientations of the smartwatch during the walking balance test. X-axis is the right (−) and the left (+) side of the participant. Y-axis is the up (−) and the down (+) of the participant. Z-axis is the forward (+) and the backward (−) of the participant.

Balance is conventionally defined as the ability to maintain the center-of-gravity (COG) of the body within its base-of-support (Hamilton, Weimar, & Luttgens, 2008). The new real-time mHealth system developed in the present study monitors constant changes of angles of the COG (defined as the middle of the waistline [umbilicus]) relative to the base-of-support. A smartwatch (LG G Watch R) is secured at the COG location of an individual during the test (see Figure 1), and its different sensors collect motion data. The rotational vector sensor in the wearable device’s software is particularly versatile and can be used to monitor a wide range of motions or movements—for instance, to detect gestures or to monitor angular or relative orientation changes. Compared to other types of sensors, the proposed rotation vector sensor may be a better choice for motion detection and monitoring, in that its represents the rotation of the device in the physical world (Incel, 2015; Sabatini, 2011). In our mHealth system, the rotation vector represents the orientation of the COG as a combination of an angle and an axis, in which the body rotates through angle *θ* around axes *x*, *y*, or *z*. Hence, in our real-time mHealth system, the constant changes of angles of the COG, deemed to be key indicators of balance performance, can be calculated based on three rotation vectors (i.e., right to left, up to down, and forward to backward; see Figure 1).

**Figure 2:**
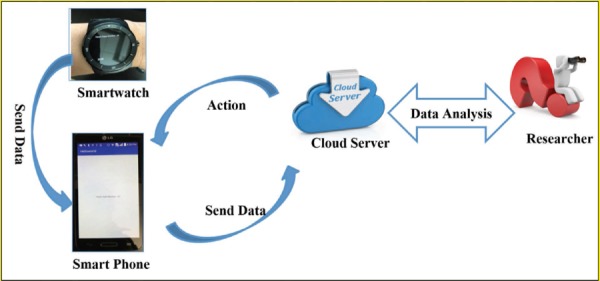
Overview of the Real-Time mHealth System.

**Figure 3:**
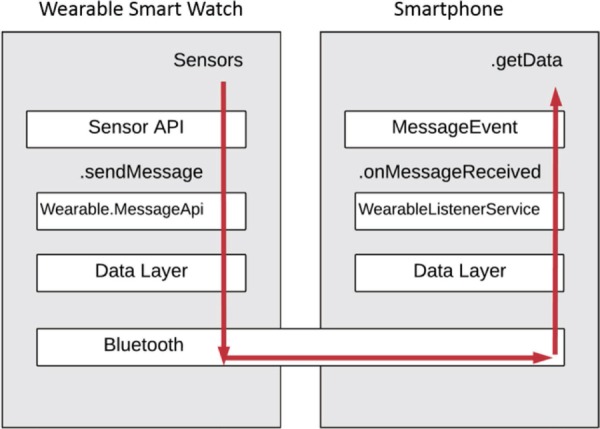
Data and program flow between the wearable smart watch and the smartphone.

For the real-time data collection and storage, we developed Android smartphone and smartphone applications. The data was transferred in real time from the smartwatch to the cloud server. The cloud server, ThingSpeak^™^ (“IoT Analytics - ThingSpeak Internet of Things,” n.d.), stores and analyzes data (see Figure 2). The proposed mHealth system consists of the following three data flow components:

- *Data Collection*. Data collection in the proposed mHealth system unfolds in two stages: data collection (Stage 1) and data storage (Stage 2). In Stage 1, the data collection module is operated by the smartwatch with an installed Android application that we developed for collecting and processing data in real time (see following section, “Android Application Development”). The Android Sensor Application Programming Interface (API) and Wearable API were used to collect the sensor data in the wearable smartwatch (see Figure 3). In Stage 2, the collected data was stored in the ThingSpeak^™^ cloud server. However, since the ThingSpeak^™^ cloud server is not suitable to collect data in real time, the collected data was first temporarily saved on the smartphone in a block size and then sent to the cloud server.
- *Cloud Server*. ThingSpeak^™^ service, an Internet-of-Things (IoT) analytic service platform to aggregate, visualize, and analyze live data streams in the cloud, was used to store the data on the cloud server. The cloud server can perform various actions, such as sending a warning message via text and/or an email to an end user and/or a researcher when the sensor data exceed the threshold point. The threshold point to set an action on the server can be replaced.
- *Research Application*. Sensor data can be downloaded from the server to produce visualization graphs (Lee, Jo, & Kim, 2014) via the cloud server. The data can also be analyzed with the tools available on the server, or with other relevant tools, such as MATLAB. In addition, other relevant clinical data can be uploaded to the server for further analysis with the mHealth data.

#### Android Application Development

The development environment for our real-time mHealth system is described in Table 1. Android applications for the smartwatch and for the smartphone were developed to collect motion data from sensors for different test participants. The following motion sensors in the smartwatch were included for data collection: the uncalibrated gyroscope, gyroscope, uncalibrated rotation on vector, accelerometer, and linear acceleration. The collected data was stored as test files in the comma-separated value (CSV) format on the smartphone. The Android application uses the Sensor API, Message API, and Wearable API. The Sensor API manages the sensors in the smartwatch. The Android smartwatch program uses the SensorManager class in the Sensor API to assess the device’s sensors: the Sensor class in Sensor API to get the list of available sensors, and the SensorEventListener class in the Sensor API to obtain sensor data. The Wearable API sends the message from the smartwatch to the smartphone via Bluetooth (see Figure 3). In the Android smartphone program, the WearableListenerService class in Wearable API receives messages from the smartwatch. The Message API in the Android smartphone program sends the data from the smartphone to the ThingSpeak^™^ server.

**Table 1:** Development Environment

Component	Program used
Client application	Android program (smartphone and smart watch)
Service server	ThingSpeak^™^
Motion sensors collecting data	Uncalibrated gyroscope, gyroscope, uncalibrated rotate on vector, accelerometer, and linear acceleration
Data format	Transformed the comma-separated value (CSV)
Application Programming Interface (API)	Sensor API
	Message API
	Wearable API

The flow of the program and data in the developed Android applications is schematically shown in Figure 3. First, the smartwatch collects the sensor data using the Sensor API. Second, the sensor data are sent to the smartphone in real time via the wearable message API and Bluetooth. Third, the smartphone receives the data using the WearableListenerService class in the Wearable API. Fourth, in the smartphone, the sensor data are transformed to the CSV format. Fifth, the formatted data stored on the smartphone are sent to the cloud server via the Message API. Finally, the server displays and saves the data on the web-server.

### Data Collection

The mHealth system was tested with a total of four participants. Two participants (F1 and F2) were Asian females aged 22 and 41 years old, respectively. Two further participants (M1 and M2) were White males aged 26 and 68 years old, respectively. One male participant (M2) had reduced physical mobility due to knee osteoarthritis and a subsequent knee replacement surgery; the other three participants did not have any known histories or problems that may have affected their postural control. Reduced mobility due to knee osteoarthritis is reported to be similar with the one observed in patients with persistent mTBI who, despite ambulatory treatment, can have balance impairments reducing their full engagement in community living (Takacs, Garland, Carpenter, & Hunt, 2014).

During the experiment, all participants wore the smartwatch in a pocket of a belt at the center of their body. The smartwatch was placed facing front, with its top facing the right side of the body (see Figure 1). The participants were instructed to walk straight on their pace at an indoor hallway of a building. The walked distance was ca. 440 feet in length. Each participant had a round trip to walk back and forth in the hallway. To eliminate interruptions, the walking sessions were organized at the time when the building was not used by other people. The duration of walking with the smartwatch ranged from 6 to 9 min.

Using the real-time mHealth system, the smartphone stored the motion sensor data on the phone and sent them to the cloud server in real time (see Figure 2). Android programs in our system collected the motion sensor data from the smartwatch and relayed them to the cloud server (see Figure 3). The mHealth system collected the sensor data of 250 ms and sent them to the cloud server at regular intervals (every minute).

### Data Analysis

**Figure 4:**
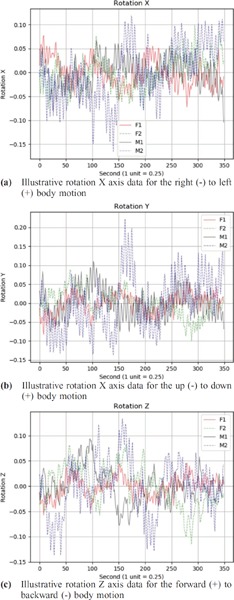
Illustrative rotation data by each axis recorded by the smartwatch from healthy individuals and the participant with reduced physical mobility. F1 and F2 denote healthy females; M1 denotes a healthy male; M2 denotes a male with reduced physical mobility. Compared to the other participants, M2 has more variations in the rotations x, y, and z, suggesting that M2 has a wider range of motion between right-and-left, up-and-down, and forward-and-backward while walking.

**Figure 5:**
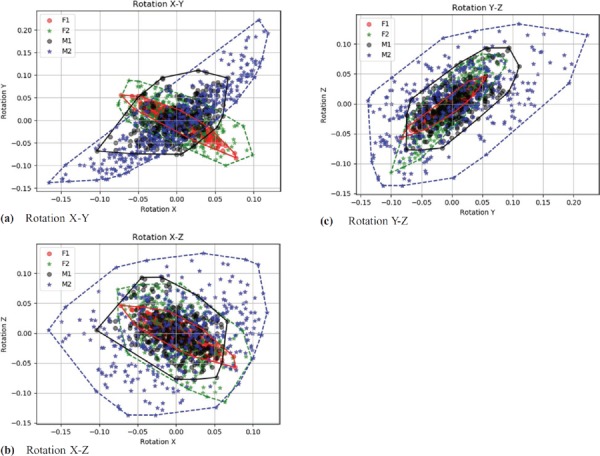
Illustrative rotation data by two combined axes during the same time period recorded by the smartwatch from three healthy individuals and one with reduced physical mobility. Illustrative rotation *X*-axis data recorded for the right (−) to left (+) body motion on the *X*-axis. Illustrative rotation *Y*-axis data recoded for the up (−) to down (+) body motion on the *Y*-axis. Illustrative rotation *Z*-axis data recoded for the forward (+) to backward (−) body motion on the *Z*-axis. F1 and F2 denote healthy females; M1 denotes a healthy male; M2 denotes a male with reduced physical mobility. (a) Female participants (F1 and F2) appeared to have narrower ranges of the right-to-left and up-and-down motions than their male counterparts (M1 and M2), with M2 having the widest range of motion while walking. (b) M2 has the widest area, suggesting that he has the widest combined motion range of right-to-left and forward-to-backward while walking. (c) M2 has the widest area, which means that he has the widest combined motion range of up-and-down and forward-to-backward while walking.

Considering that, for the differentiation of balance control during walking across different participants, the rotation vector data are considered to be the most effective (Incel, 2015; Sabatini, 2011), in the present paper, only the rotation vector data was used for the analysis. This data was extracted using the rotation sensor in Android Open Source Project (AOSP) to compare the performance of the four study participants. Using the smartwatch, the XYZ-axis rotation vectors of the belly button were identified as the COG of each participant. *X*-axis represents the body motion angle between the right and the left side of a participant. *Y*-axis represents the body motion angle between the up and the down of a participant. *Z*-axis represents the body motion angle between the forward and the backward of a participant (see Figure 1). Although each participant wore the smartwatch on the same location of his/her body, the sensors in the smartwatch appeared to be located with slightly different slopes. Therefore, for the data analysis, we standardized the data by using each differential from the average (deviation or variation) for each of the rotation vectors (see Table 2). For a comparison across the participants, the rotation vector values were plotted as 2D graphs (see Figures 4 and 5).

**Table 2:** Comparison of Participants’ Rotation Vector Data by Each Axis

Participant ID	Balance orientation axis	Max	Min	Average	Variance	STD
F1	*x*	0.077	−0.072	−5.646	0.00075	0.027
	*y*	0.055	−0.083	−4.726	0.00063	0.025
	*z*	0.046	−0.057	−4.044	0.00038	0.019
F2	*x*	0.099	−0.076	−5.456	0.00108	0.032
	*y*	0.088	−0.100	−5.837	0.00111	0.033
	*z*	0.083	−0.115	−1.269	0.00154	0.039
M1	*x*	0.067	−0.104	−6.217	0.00094	0.031
	*y*	0.110	−0.075	−1.142	0.00147	0.038
	*z*	0.094	−0.077	−3.489	0.00107	0.033
M2	*x*	0.119	−0.166	−7.613	0.00366	0.061
	*y*	0.222	−0.138	−3.045	0.00547	0.074
	*z*	0.134	−0.137	−2.379	0.00281	0.053

*Note*. The table shows minimum (standardized), maximum (standardized), average, variance, and standard deviation of each participant’s data by each axis. *X*-axis is the right (−) and the left (+) side of the participant. *Y*-axis is the up (−) and the down (+) of the participant. *Z*-axis is the forward (+) and the backward (−) of the participant. F1 and F2 = healthy females; M1 = a healthy male; M2 = a male with reduced physical mobility.

## Results

We collected constant motion data using different sensors listed in Table 1. Thereafter, to understand constant motion changes in the middle of the participants’ waistline (umbilicus) as the COG, the rotation vector values of four participants were extracted and compared (see Table 2).

The results showed that there were differences in the body motion data between the participant with reduced physical mobility (M2), on the one hand, and the healthy participants (F1, F2, and M1), on the other hand. Specifically, M2 appeared to have more variations in all rotations (*x*, *y* and *z*) than the healthy participants, suggesting that he had a wider range of motion between right and left, up and down, and forward and backward while walking (see Figures 4(a)–(c)).

For further comparisons between the participants, we also tried to combine 2 axes at a time (see Figure 5(a)–(c)). Regarding the comparison of the participant with reduced physical mobility (M2), and the healthy participants (F1, F2, and M1), we found that M2 had the widest combined motion range of right-to-left and forward-to-backward (see Figure 5(b), as well as up-and-down and forward-to-backward (Figure 5(c)). Interestingly, our results also highlighted gender-based divergences: specifically, as shown Figure 5(a), the female participants (F1 and F2) appeared to have narrower ranges of right-to-left and up-and-down motions as compared to their male counterparts (M1 and M2), with M2 having the widest range.

## Discussion

In the present study, constant motion data using the mHealth system were collected in real time from four participants; three female and male non-disabled adults (F1, F2, and M1) and one male adult with reduced physical mobility (M2). Previous studies using wearable sensor-based balance performance tests have mostly focused on measuring and analyzing data from disabled or elderly adults, without comparing the performance of these patients against normal healthy adult controls (Gordt, Gerhardy, Najafi, & Schwenk, 2018). By contrast, the present study sought to acquire a scalable range of detecting deficits in postural control among individuals with a suspected mTBI. To this end, we tested our real-time mHealth system first with healthy male and female adults and then compared their performance to that of the individual with reduced physical mobility (M2). Our results data measured by the mHealth system showed that, compared to healthy individuals, M2 had a wider range of motion between right and left, up and down, and forward and backward while walking, which resembles the diminished standing balance pattern observed after mTBI—more or wider body sway with larger oscillations (Degani et al., 2017; Gera, Chesnutt, Mancini, Horak, King, 2018). Therefore, our findings from the proof-of-concept testing validate the proposed mHealth system as a promising application for the mTBI population. Another promising application of the proposed real-time mHealth system relates to capturing early indicators of neurological deficiencies post-injury. Previously, impairment in postural control during gait was reported to be detected as early as one-month post-injury or years after an mTBI or concussion (Buckley et al., 2016; Kleffelgaard, Roe, Soberg, & Bergland, 2012; Parker, Osternig, van Donkelaar, & Chou, 2006). The proposed system capable of detecting postural control deficits in real time can provide meaningful data in this respect.

Furthermore, our results highlighted several gender-related differences. Specifically, female participants were found to have narrower ranges of right-to-left and up-and-down motions than their male counter-parts. This finding is largely consistent with previous findings. For instance, gender-related differences in walking speed and/or gait were found among a retired community (Inoue et al., 2017) and among patients with multiple sclerosis (Pau et al., 2017). Furthermore, several differences in static and dynamic postural balance differences were observed among young male and female dancers (Steinberg, Adams, Waddington, Karin, & Tirosh, 2017). Another study that investigated gender differences in gait among adolescents shortly after sports-related concussions reported that, while both concussed female and male groups showed a slower gait than the healthy control group, the post-injury impact on gait was greater in females than in males (Howell, Stracciolini, Geminiani, & Meehan, 2017). Specifically, the difference in walking cadence between the concussed and healthy females was larger than the one among males (Howell et al., 2017). In the context of these findings, our finding that female participants had narrower ranges of right-to-left and up-and-down motions than their male counterparts demonstrates that the proposed mHealth system can effectively capture gender-related differences in motion changes during walking. In future research aiming to identify the potential impact of gender in deficits of balance control after an mTBI, more data will need to be collected, particularly from the mTBI group as compared to healthy controls.

Next, while the proposed mHealth system enabled us to collect data from the uncalibrated gyro-scope, gyroscope, accelerometer, and linear acceleration sensors using the Android program for the smart-watch, in the analyses, only the rotation vector data was mainly used. This methodological decision was made because the rotation vector data have been reported to be the most effective in terms of differentiating balance control during walking across different participants (Incel, 2015; Sabatini, 2011). While other types of sensors in the wearable devices may also present clinically meaningful data or parameters for balance control, relevant studies using other sensors remain scarce. Therefore, in further research, it would be necessary to identify better approaches to the analysis of those sensors’ data with a bigger sample size, including individuals with different levels of physical mobility levels. In combination with further data from those sensors, our rotation vector data will contribute to advancing our real-time mHealth system to better detect early signs and symptoms of deficits in balance control caused by mTBI.

Currently used objective measures of balance performance for mTBI, such as a force-platform postural-stability test, a motion capture system, and a sensory-motor measurement system, are mostly supervised and lab-based. With an emphasis on portability, the Nintendo Wii Balance Board (Nintendo of America Inc., Redmond, WA), originally designed as part of a video-game console system, has also been used to assess the postural center-of-pressure in athletes (Merchant-Borna et al., 2017). However, all these approaches require supervising the individuals affected by mTBI in clinical or research laboratory settings, which may hinder time-sensitive symptom assessment, as individuals affected by mTBI may not seek proper screening or not recognize their symptoms post-injury (Seabury et al., 2018). From the ethnic perspective, evidence is available showing that several Asian groups, including Pacific Islanders (Tauafiafi, 2014), have a higher incidence rate and poorer outcomes of TBIs, including mental health post-injury, as well as a lower compliance in home-based treatment, compared to non-Hispanic Whites (Dams-O’Connor et al., 2013; McQuistion et al., 2016; Perrin et al., 2014; Staudenmayer et al., 2007). In view of these findings, our new real-time mHealth system can offer a possibility of reliable and accurate home-based monitoring for racial minority groups who have had an mTBI. In addition, the use of the proposed system will also contribute to initiating timely and appropriate treatment and to a more accurate evaluation of the rehabilitation process.

## Limitations and Conclusions

The present study has several limitations. Since our major goal was the proof-of-concept test of the newly developed real-time mHealth system, including establishing the necessary data analysis steps, we have not generated clinically meaningful data yet. Nonetheless, as suggested by our results, the proposed real-time mHealth system can effectively capture differences in postural control during walking in healthy individuals vs. individuals with reduced physical mobility, as well as in female vs. male participants. In further research, we will conduct feasibility and efficacy testing with larger pools of individuals with mTBI, along with data security planning for the real-time data transmission and storage. In addition, any variables that may affect postural balance during walking, such as body habitus (e.g., slim vs. obese) or level of daily physical activities, will be taken into account in data collection and analysis. We will also consider evaluating the impact of the application at the health system level, using outcomes such as health care utilization and medication use.

In this proof-of-concept testing, we performed the unsupervised real-time measurement of walking balance using the mHealth system. In the proposed mHealth system, a smartwatch, a smartphone, and a cloud server communicate together to measure, collect, and store body balance data in real time. Taken together, our results demonstrated the feasibility of home-based self-management for mTBI symptoms via the mHealth approach and suggest that this system can meaningfully complement conventional mTBI treatments. Using the mHealth system presented in this study, individuals with suspected mTBI can conveniently receive real-time feedback on their data in their home environment and interact with their health care providers and/or researchers. On the condition of patient engagement, the proposed system also offers early detection of neurological deterioration after mTBIs, which can yield better-quality outcomes in health care.

In sum, through the remote real-time assessment of mTBI symptoms, the proposed approach contributes to enhancing home-based symptom management for individuals with mTBI. In the future, more research on both individuals with mTBI and healthy controls is necessary to validate, refine, and apply our mHealth system for monitoring postural balance during walking after mTBI.

## Declaration of Conflicting Interests

The authors declared no potential conflicts of interest concerning the research, authorship, or publication of this article.

## Funding

This study was supported by a grant from the UNLV School of Nursing and the UNLV Faculty Top Tier Doctoral Graduate Research Assistantship.
